# Hyperacute T Waves Are Specific for Occlusion Myocardial Infarction, Even Without Diagnostic ST-Segment Elevation

**DOI:** 10.1016/j.jacadv.2025.102120

**Published:** 2025-08-31

**Authors:** H. Pendell Meyers, František Simančík, Robert Herman, Adam Rafajdus, William H. Frick, José Nunes de Alencar, Emre K. Aslanger, Stephen W. Smith

**Affiliations:** a Department of Emergency Medicine, Carolinas Medical Center, Charlotte, North Carolina, USA; b Powerful Medical, Bratislava, Slovakia; c Cardiovascular Center Aalst, OLV Hospital, Aalst, Belgium; d Department of Cardiology, SSM Health St. Louis University Hospital, St. Louis, Missouri, USA; e Instituto Dante Pazzanese de Cardiologia, São Paulo, Brazil; f Department of Cardiology, Basaksehir Pine and Sakura City Hospital, Health Sciences University, Istanbul, Türkiye; g Department of Emergency Medicine, Hennepin Healthcare, University of Minnesota, Minneapolis, Minnesota, USA

**Keywords:** acute coronary syndrome, hyperacute T-waves, occlusion myocardial infarction, ST-segment elevation myocardial infarction equivalent, ST-segment elevation myocardial infarction

## Abstract

**Background:**

Despite no objective definition, hyperacute T waves (HATWs) are recommended by the American College of Cardiology as a ST-segment elevation myocardial infarction (STEMI) equivalent finding, requiring emergent reperfusion.

**Objectives:**

We sought to derive and validate a quantitative definition of HATW.

**Methods:**

We retrospectively evaluated adults with possible acute coronary syndrome across five percutaneous coronary intervention centers. Exclusions were lack of preangiogram ECG, QRS duration ≥110 ms, and elevated troponin without angiogram. The outcome definition was myocardial infarction with TIMI flow grade 0-1 culprit lesion. ECG measures included T-wave magnitude (T-wave area relative to QRS amplitude) and symmetry (T-wave peak-to-end time relative to onset-to-peak time). The HATW score was derived and evaluated on separate groups. The primary analysis was HATW score performance for acute coronary occlusion in patients without STEMI criteria.

**Results:**

3,274 patients were reviewed, and 618 were excluded. 1,261 and 1,395 patients were allocated to derivation and validation groups. In derivation, the optimal HATW score threshold for ≥98% specificity was 2 consecutive leads with mean HATW score ≥0.7. In validation, the performance for acute coronary occlusion in the subset without STEMI criteria (N = 1,300) was 98.4% specificity, 20.7% sensitivity, 47.4% positive predictive value, and 12.54 positive likelihood ratio. Among patients without STEMI criteria but positive HATW score, 84% had a culprit lesion causing acute myocardial infarction.

**Conclusions:**

The HATW score is the first objective definition of HATW showing significant clinical utility as an ECG finding of acute coronary occlusion in potential acute coronary syndrome patients.

Hyperacute T-waves (HATWs) are widely recognized as a subtle yet significant electrocardiographic marker of acute coronary occlusion (ACO) myocardial infarction (MI)—increasingly known as occlusion myocardial infarction (OMI).^1^ It has been shown that HATWs can diagnose^2^^,^^3^ and localize^4^ ACO earlier than and with specificity comparable to standard ST-segment elevation myocardial infarction (STEMI) criteria. Accordingly, both the American College of Cardiology (ACC)^5^ and European Society of Cardiology (ESC)^6^ have recommended identifying HATWs as a STEMI equivalent, requiring emergent reperfusion therapy.

Although subjective identification of HATW by electrocardiogram (ECG) experts has demonstrated their diagnostic utility, there have been no objective definitions or criteria proposed. Prior attempts to study an objective definition of HATWs have been limited by inadequate definitions of HATW and by methodology that renders the data inapplicable for the study of ACO. Koechlin et al attempted to define HATWs only by T-wave amplitude and found no association between T-wave amplitude and clinical diagnosis of acute MI,^6^^,^^7^ but there were significant methodological problems.^8^ Collins et al showed that a ratio of T-wave amplitude to QRS amplitude of >75% was a predictor of acute MI, though did not distinguish acute MI caused by ACO (requiring emergent reperfusion) from other MI (not requiring emergent reperfusion).^9^ Hochrein et al studied isolated T-wave amplitude in the same leads as STE in the Global Utilization of Streptokinase and Tissue Plasminogen Activator for Occluded Coronary Arteries trial to evaluate the hypothesis that “high” T-waves (>98th percentile of the study population) would have prognostic significance as an early ECG finding in MI.^10^ Indeed, the investigators found that patients with STEMI and high T-waves who received thrombolytics had faster time to treatment, lower 30-day mortality, and lower rates of heart failure and cardiogenic shock as compared to patients without high T-waves.

Considering the limitations of abovementioned definitions, we sought to derive and validate a quantitative definition of HATWs and evaluate its performance as a diagnostic indicator for ACO in a cohort of possible acute coronary syndrome (ACS) patients in the emergency department (ED), specifically in patients whose ECGs do not meet STEMI criteria.

## Methods

### Study design and population

We retrospectively evaluated data from adults (age ≥18 years) undergoing ED assessment for possible ACS across five percutaneous coronary intervention (PCI) centers, including sites in Belgium, Italy, Lebanon, and 2 in the United States. The data collection methods have been described previously.^2^^,^^11^ Study approval was obtained from each participating site’s Institutional Review Board. Briefly, adult patients presenting to the ED with a clinical picture of ACS were included. Patients without a preangiogram ECG, those with a wide QRS complex (QRS duration ≥110 ms), and patients with elevated troponin but no angiographic data (for whom the primary outcome cannot be judged) were excluded. Wide QRS complex morphology was excluded because the associated secondary repolarization abnormality distorts T-wave morphology which may confound results and thus warrant a separate study. Clinical details were collected via electronic chart review for each patient, including demographics, troponin, and angiogram results during the index presentation. According to the U.S. Food and Drug Administration’s best practice recommendations for machine learning, training (derivation), and test (validation) data sets should come from different sites.^12^ Thus we reserved the center with the most patients (n = 1,395) for the validation group, and the remaining patients (n = 1,261) were assigned to the derivation group.

### Clinical outcome measures

#### Primary definition of OMI

An acute culprit lesion with TIMI flow grade 0 or 1, as determined by the treating interventional cardiologist during angiography.

#### Secondary definitions of OMI

1. A culprit lesion with TIMI flow grade 0 or 1, or TIMI flow grade 2 or 3 accompanied by a peak troponin T ≥1,000 ng/L.
2. Expert contextual annotation of OMI via thorough chart review of all clinical information (judged by researchers H.P.M. and S.W.S.).

These definitions have been used previously, and their advantages and limitations (especially considering that a significant portion of true STEMI patients have an open culprit artery by the time of angiogram) have been explained in detail.^2^ Additionally, we performed an additional exploratory analysis using “acute MI with PCI” as a possible outcome definition of interest.

### ECG measures

The first available ECG on presentation before angiogram was used for the primary analysis. All ECGs were reviewed by 2 experts and proprietary software (PMcardio by Powerful Medical, Bratislava, Slovakia) that has been used in prior publications.^11^^,^^13^ PMCardio was used for automatic construction of median beats and measurements of all ECG features, including amplitudes, area under the curve, and duration of waves, as well as measurement of STEMI criteria per the Fourth Universal Definition of Myocardial Infarction (STE at the J-point relative to the QRS onset).^14^ Interrater reliability for STEMI criteria determination was performed between ECG experts (H.P.M. and S.W.S.) and computer automated STEMI measurements.

We have previously contended that HATWs are not objectively defined by their amplitude, but rather by the following features: large “bulk,” defined as high area under the T-wave relative to the QRS amplitude, increased T-wave symmetry, and increased T-wave convexity.^8^ Thus, we defined hyperacute T-wave features in this study as T-wave magnitude and T-wave symmetry (see Central Illustration). T-wave magnitude is measured by area under the curve from the J point to the end of the T-wave, relative to the total QRS amplitude (measurement from maximum to minimum points of the QRS). T-wave symmetry is measured by the time from T-wave peak to T-wave end relative to the time from the J point to the T-wave peak. Both the magnitude and symmetry score are calculated by logistic regression from the described measurements above, resulting in a unitless number between 0 and 1.

**Central Illustration:**
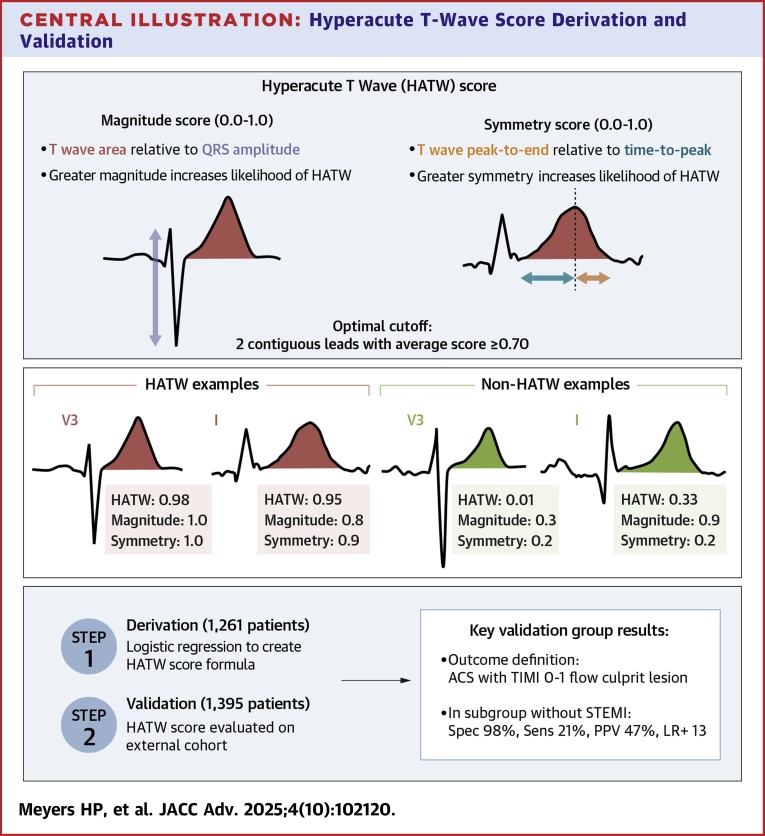
Hyperacute T-Wave Score Derivation and Validation (Upper left) Explanation and demonstration of T-wave magnitude and symmetry. (Upper right) Examples of hyperacute (red) vs normal (green) T-waves. (Bottom) Key study design and outcome information. Abbreviations as in Figures 2, 3, and 5.

In the derivation group, ECG experts who have already shown high accuracy in the recognition of HATW as markers of OMI (H.P.M. and S.W.S.)^2^ subjectively evaluated all ECGs for HATWs, with all available outcome data (angiography, serial ECGs, troponins, etc) available for review. Presence or absence of HATWs was marked in each lead, and each HATW was rated on a scale of 0 to 5: 0 meaning not hyperacute, 1 being the most subtle HATW, and 5 being the most obvious and most diagnostic HATW. To prevent inclusion of unrecognized subtle HATWs in the control group, ECGs containing one or more expert-annotated HATW in any lead were considered ineligible to serve as control (ie, non-HATW) for any of the 12 leads. See Supplemental Figure 1 for an example of an ECG in the derivation group with each lead annotated for HATWs.

### HATW score development (derivation group)

The primary aim of the derivation group was to derive a HATW score by logistic regression, composed of lead-specific coefficients for T-wave magnitude and symmetry, to optimally match the identification of expert-annotated HATWs. Lead aVR was excluded, as we deem it not applicable to have upright, HATW in aVR, assuming normal anatomy and lead placement.

Individual ECG leads are only eligible for the HATW score if they satisfy the following requirements:

1. Not lead aVR
2. Narrow QRS: QRS duration ≤110 ms
3. Well-defined QRS complex: Total QRS amplitude ≥1.0 mm (0.1 mV)
4. Well-defined and positive T-wave:
  a. Positive T-wave amplitude ≥1.0 mm (0.1 mV)
  b. TW start-to-peak and TW peak-to-end each ≥40 ms
5. T-wave neither inverted nor biphasic:
  a. From T-wave start to peak, waveform may not descend below 0.5 mm (0.05 mV) from start
  b. From T-wave peak to end, waveform may not descend below 1.0 mm (0.10 mV) from end

A secondary aim of the derivation group was to compare the performance of the T-wave magnitude (T-wave area/QRS amplitude) against potentially simpler measurements (eg, simple T-wave amplitude). Finally, from prior experience, we believed that HATW characteristics differ among different lead groups (eg, limb leads, precordial leads). Thus, the analysis was divided into 3 groups: limb leads, V_1_-V_3_, and V_4_-V_6_.

### HATW score validation (validation group)

The primary aim of the validation group was to evaluate the diagnostic performance of the derived HATW score for the diagnosis of OMI on a separate cohort of suspected ACS patients. The primary analysis was defined as diagnostic accuracy statistics (eg, sensitivity, specificity) of the HATW rule for OMI, specifically in the subset of patients without STEMI criteria. Secondary analyses included comparison of diagnostic accuracy of HATW score vs STEMI criteria among all patients (including those with STEMI criteria), and evaluation of timing from presentation to intervention for key subgroups.

### Statistics

#### HATW score model training

The HATW score model was trained as a small 2-layer feedforward neural network with logistic activation function. The architecture is demonstrated in Supplemental Figure 2. The first hidden layer consists of 2 neurons computing the intermediate magnitude and symmetry scores, respectively. The second output layer combines the intermediate scores into the final HATW score output. We used standard techniques for neural-network training to optimize all parameters of the model in unison.

Expert-annotated HATW leads were considered positive HATW leads for the model training, and each lead was weighted by the 1 to 5 severity rating provided by the annotators. Control leads (those without HATW) were considered negative for model training with the weight of 1. In order to prioritize and optimize the performance of the HATW score in the subgroup of patients without STEMI criteria, any leads from STEMI-criteria ECGs were excluded from the model training. The model was trained to minimize the binary cross-entropy loss for binary classification, using the AdamW^15^ optimizer with the learning rate of 0.004, weight decay of 0.05, and 20,000 training epochs.

## Results

**Figure 1:**
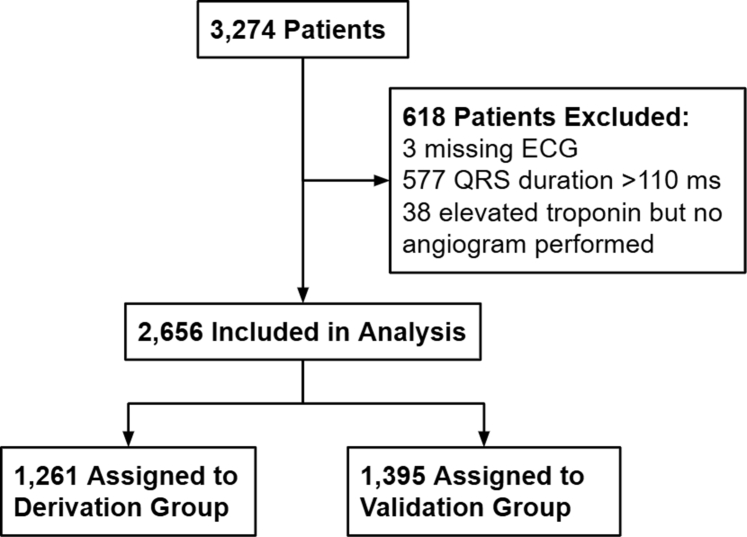
Patient Inclusion and Exclusion Flowchart A total of 2,656 pts from 5 sites across 3 continents were included, then assigned to derivation and validation groups.

A total of 3,274 patients were available from all 5 sites, of which 3 were excluded for missing preangiogram ECG, 577 excluded for QRS duration ≥110 ms, and 38 were excluded for elevated troponin without angiogram performed, thus the final study population consisted of 2,656 patients (see Figure 1). As explained in methods, 1,395 patients were reserved for the validation group, and the remaining 1,261 patients were assigned to the derivation group.

### Derivation results of the HATW score

The derivation group included 2,079 ECGs from 1,261 patients, with 21% (437/2,079) prevalence of OMI and 7.4% (153/2,079) prevalence of STEMI(+) OMI. Average (SD) age was 56.7 (15.9) years, and 59% were male. All ECGs were evaluated for STEMI criteria by both experts and automated PMCardio STEMI criteria algorithm, resulting in 97.3% agreement (Cohen’s κ = 0.80).

Expert annotations yielded 1,401 total leads with HATWs and 18,062 without (interrater reliability statistics are presented in Supplemental Figure 2). Supplemental Table 1 shows the number of HATW and control T-waves in each lead. Of note, there were insufficient HATW annotations in lead V_1_ to include lead V_1_ in the logistic regression, so V_1_ was excluded from further analysis and from the derived HATW score. Supplemental Table 2 compares the HATWs and control group T-waves in terms of T-wave amplitude, T-wave area, and the ratio of T-wave area to QRS amplitude. All measurements of correlation and all diagnostic accuracy statistics were significantly greater using the ratio of T-wave area to QRS amplitude rather than simple T-wave amplitude alone. As such, it was not possible to derive a clinically useful HATW rule or score based only on simple T-wave amplitude, to achieve 98% specificity and >10% sensitivity.

**Figure 2:**
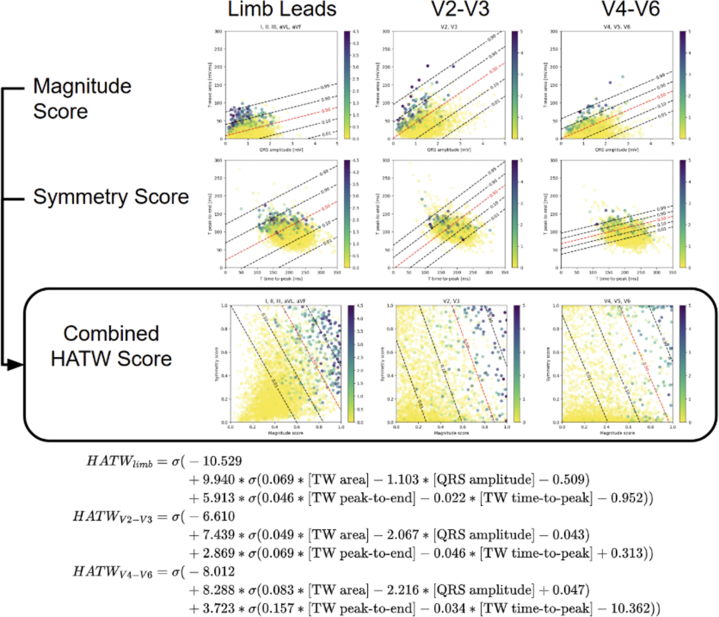
Hyperacute T Wave Score Development (Upper) T-wave magnitude and symmetry plots for control T-waves (yellow dots) and HATWs (green to purple dots according to expert annotated HATW severity score), as well as the overlaid logistic regression optimized to differentiate HATW from non-HATW. As shown, T-wave magnitude is a measure of T-wave area relative to QRS amplitude, and T-wave symmetry is a measure of the time from T-wave peak-to-end to the time from T-wave start-to-peak. (Lower) The resulting HATW score is shown in full equation form, in which σ is the standard logistic function defined by $\sigma \left(x\right)=1\;/\;\left(1+e^{-x}\right)$. HATW = hyperacute T-waves.

Figure 2 shows T-wave magnitude and symmetry plots for control T-waves (yellow dots) vs HATWs (green to purple dots according to expert annotated HATW severity score), demonstrating that HATWs generally have greater symmetry and magnitude than control T-waves.

At a threshold score of 0.5, the HATW score had 98.6% specificity and 57.1% sensitivity for identifying individual HATW leads as judged by ECG experts. The performance for each individual lead ranged from 97.3% to 99.5% for specificity and from 42.7% to 66.7% for sensitivity. In derivation group ECGs without STEMI criteria, when 2 contiguous leads are required to have an average HATW score of 0.7, this finding had 99.2% specificity and 27.4% sensitivity for the diagnosis of OMI (by expert reviewers with full clinical context available).

#### Optimal threshold selection

After training the per-lead HATW score, we chose an optimal per-ECG threshold targeting the maximum sensitivity achievable at a minimum 98% specificity (chosen to match the specificity of STEMI criteria in detecting OMI on the derivation group). To this end, for a positive per-ECG OMI prediction, we required 2 contiguous leads with their average HATW score of at least 0.7. At this threshold, our HATW rule had 98.7% specificity and 41.7% sensitivity on the whole derivation group (including STEMI criteria ECGs), and 99.2% specificity and 27.4% sensitivity on the derivation group excluding STEMI-criteria ECGs.

### Validation results of the HATW score

The validation group included the 1,395 initial ECGs from 1,395 potential ACS patients in the ED with 10.6% (148/1,395) prevalence of OMI (defined by acute myocardial infarction (AMI) with culprit lesion TIMI flow grade 0-1) and a 6.8% (95/1,395) prevalence of STEMI(+) OMI. Average (SD) age was 64 (12) years, and 64% were male. All ECGs were evaluated for STEMI criteria by both experts and automated PMCardio STEMI criteria algorithm, resulting in 98.4% agreement (Cohen’s κ = 0.88).

Table 1 shows characteristics and clinical outcome information for the validation group.

**Table 1:** Clinical Characteristics and Key Outcome Information for the Validation Group

	All Patients (N = 1,395)	OMI Patients (AMI + Culprit TIMI Flow Grade 0-1) (n = 148)	Patients Without OMI (n = 1,247)
Age (y)	64 ± 12	63 ± 12	65 ± 12
Female	509 (36.5%)	26 (17.6%)	483 (38.7%)
STEMI criteria present on ECG	95 (6.8%)	61 (41%)	34 (2.7%)
Coronary angiography performed during hospitalization	893 (64.0%)	148 (100%)	745 (59.7%)
Time to angiography in hours	19.2 (6.3-25.6)	2.25 (0.9-17.2)	20.1 (16.1-27.8)
PCI performed	483 (34.6%)	146 (98.6%)	337 (27.0%)

Values are mean ± SD, n (%), or median (IQR).

AMI = acute myocardial infarction; OMI = occlusion myocardial infarction; PCI = percutaneous coronary intervention; STEMI = ST-segment elevation myocardial infarction.

#### All validation group patients (with and without STEMI criteria)

Among the entire validation group, including those with STEMI criteria (N = 1,395) the diagnostic performance of the HATW score (using 2 consecutive leads with average HATW score 0.7 or greater) for the identification of OMI (TIMI flow grade 0-1 culprit) achieved 97% specificity, 45% sensitivity, 63% positive predictive value (PPV), 94% negative predictive value (NPV), 14.16 positive likelihood ratio (LR+), and 0.57 negative likelihood ratio (LR−). For comparison, STEMI criteria had 97% specificity, 41% sensitivity, 64% PPV, 93% NPV, 15.26 LR+, and 0.60 LR−. Neither sensitivity nor specificity was statistically different by McNemar’s test of paired proportions. Compared to using STEMI criteria alone (97% specificity and 41% sensitivity), using STEMI criteria and/or HATW score yielded similar specificity (96%) with increased sensitivity (53%).

#### Validation group patients without STEMI criteria

The primary analysis was the performance of the HATW score ≥0.7 (mean of 2 contiguous leads) for the identification of OMI (AMI with culprit lesion TIMI flow grade. 0-1) in the subset of patients without STEMI criteria (N = 1,300), for which the HATW score had 98.4% specificity, 20.7% sensitivity, 47.4% PPV, 94.5% NPV, 12.54 LR+, and 0.81 LR−.

Secondary analyses examined the same performance metrics using alternative definitions of OMI. Using the second possible OMI definition (AMI with culprit lesion TIMI flow grade 0-1, or TIMI flow grade 2-3 with troponin T >1,000 ng/L) resulted in 98.8% specificity, 16.3% sensitivity, 63.2% PPV, 90.3% NPV, 13.50 LR+, and 0.85 LR−. Using the third possible definition of OMI (expert chart review with all clinical data available), the HATW rule had 99.6% specificity, 30.3% sensitivity, 86.8% PPV, 94.0% NPV, 72.10 LR+, and 0.70 LR−. In our exploratory analysis with the outcome definition of AMI with PCI performed, the HATW rule had 99% specificity, 8.6% sensitivity, 73.7% PPV, 76.5% NPV, 8.37 LR+, and 0.92 LR−.

**Figure 3:**
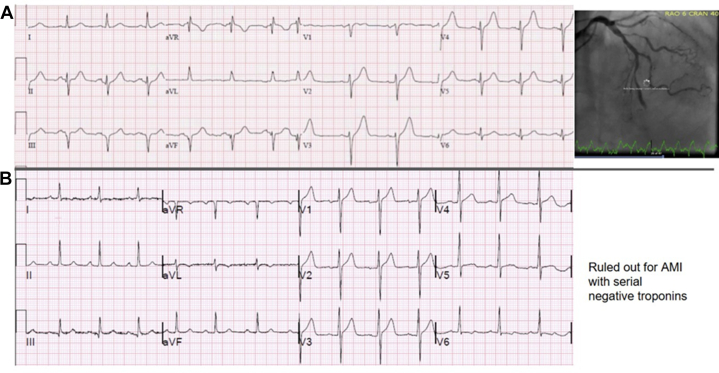
Example Cases With Hyperacute vs Normal T Waves The top ECG (A) has very little ST-segment elevation but has a relatively large T-wave area relative to QRS size in leads V_2_-V_5_, and the T-waves are very symmetric; thus, it is diagnostic of left anterior descending OMI which was confirmed on emergent angiography. The bottom ECG (B) has more ST-segment elevation, but still does not meet STEMI millimeter criteria. The T-waves have a smaller area than in (A) and the QRS amplitude is larger, thus quantified T-wave magnitude is less. The T-waves also have a slower upstroke than downstroke and thus are less symmetric, which is typical of the T-wave in normal variant ST-segment elevation. Patient B ruled out for AMI. AMI = acute myocardial infarction; OMI = occlusion myocardial infarction; STEMI = ST-segment elevation myocardial infarction.

**Figure 4:**
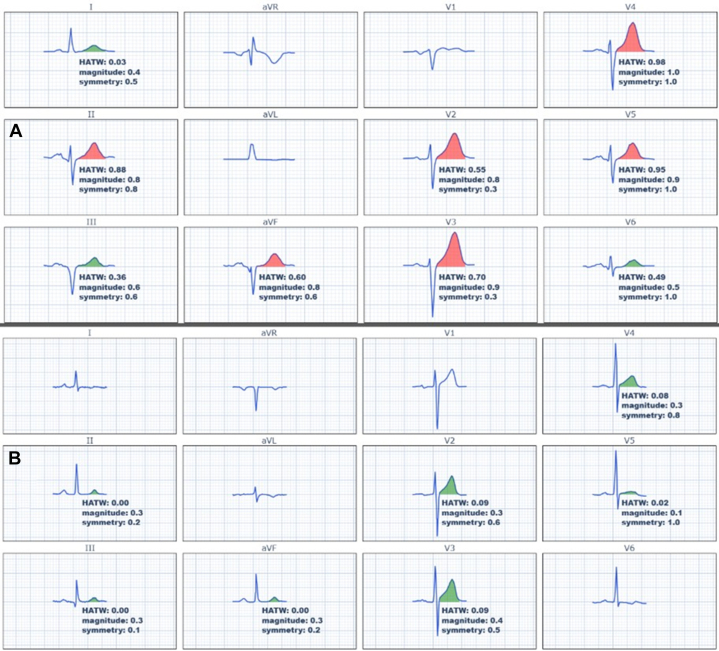
Example Cases With HATW Scores Median beat representations of the ECGs (A, B) from Figure 3, including the scores for HATW score, magnitude score, and symmetry score for each lead. Red T-wave highlighting indicates a single lead score ≥0.5, while green highlighting indicates <0.5. The magnitude, symmetry, and resulting HATW scores for these ECGs are also presented superimposed on the logistic regression diagrams in Supplemental Figure 4. Abbreviation as in Figure 2.

Table 2 shows diagnostic accuracy statistics of selected combinations of HATW score thresholds, with and without presence of STEMI criteria, for each outcome definition studied. Supplemental Table 3 shows all studied combinations. Figures 3 and 4 show relevant example cases.

**Table 2:** Diagnostic Accuracy Statistics of Various HATW Score Thresholds, With and Without Presence of STEMI Criteria, for Each Outcome Definition Studied

Score threshold (2 contiguous leads with)	Primary OMI Definition (AMI With Culprit Lesion With TIMI Flow Grade 0-1) (148 With OMI, 1,247 Without OMI)																	
	All Patients (N = 1,395)						STEMI Criteria (+) Patients (n = 95)						STEMI Criteria (−) Patients (n = 1,300)					
	Spec	Sens	PPV	NPV	LR+	LR−	Spec	Sens	PPV	NPV	LR+	LR−	Spec	Sens	PPV	NPV	LR+	LR−
Mean ≥0.5	94.2	46.6	48.6	93.7	7.97	0.57	32.4	82.0	68.5	50.0	1.21	0.56	95.9	21.8	27.5	94.5	5.30	0.82
Mean ≥0.6	95.4	46.0	54.4	93.7	10.05	0.57	38.2	80.3	70.0	52.0	1.30	0.51	97.0	21.8	34.6	94.5	7.35	0.81
Mean ≥0.7	96.8	45.3	62.6	93.7	14.10	0.57	41.2	80.3	71.0	53.9	1.37	0.48	98.4	20.7	47.4	94.5	12.54	0.81
Mean ≥0.8	97.4	41.9	65.3	93.4	15.81	0.60	44.1	78.7	71.6	53.6	1.41	0.48	98.9	16.1	50.0	94.3	13.99	0.85

**Score threshold** **(2 contiguous leads withe)**	**Alternative OMI Definition (AMI With Culprit Lesion With TIMI Flow Grade 0-1, or Flow Grade TIMI 2-3 With Troponin T >1,000 ng/L)** **(224 With OMI, 1,171 Without OMI)**																	

	**All Patients** **(N = 1,395)**						**STEMI Criteria (+) Patients** **(n = 95)**						**STEMI Criteria (−) Patients** **(n = 1,300)**					
**Spec**	**Sens**	**PPV**	**NPV**	**LR+**	**LR−**	**Spec**	**Sens**	**PPV**	**NPV**	**LR+**	**LR−**	**Spec**	**Sens**	**PPV**	**NPV**	**LR+**	**LR−**

Mean ≥0.5	95.8	41.5	65.5	89.6	9.93	0.61	50.0	83.1	87.7	40.9	1.66	0.34	96.5	19.7	42.0	90.4	5.69	0.83
Mean ≥0.6	96.9	39.7	71.2	89.4	12.94	0.62	50.0	79.2	87.1	36.0	1.58	0.42	97.7	19.1	50.9	90.4	8.14	0.83
Mean ≥0.7	98.1	38.0	79.4	89.2	20.19	0.63	55.6	79.2	88.4	38.5	1.78	0.37	98.8	16.3	63.2	90.3	13.50	0.85
Mean ≥0.8	98.6	35.3	83.2	88.9	25.74	0.66	61.1	77.9	89.6	39.3	2.00	0.36	99.2	12.9	67.9	89.9	16.58	0.88

**Score threshold** **(2 contiguous leads with)**	**Alternative OMI definition (expert chart review) (199 with OMI, 1,196 without OMI)**																	

	**All Patients** **(N = 1,395)**						**STEMI Criteria (+) Patients** **(n = 95)**						**STEMI Criteria (−) Patients** **(n = 1,300)**					
**Spec**	**Sens**	**PPV**	**NPV**	**LR+**	**LR−**	**Spec**	**Sens**	**PPV**	**NPV**	**LR+**	**LR−**	**Spec**	**Sens**	**PPV**	**NPV**	**LR+**	**LR−**
Mean ≥0.5	97.7	57.3	80.3	93.2	24.48	0.44	80.0	80.0	98.6	18.2	4.00	0.25	97.7	38.5	60.9	94.6	16.97	0.63
Mean ≥0.6	98.7	54.8	87.2	92.9	40.87	0.46	80.0	76.7	98.6	16.0	3.83	0.29	98.7	36.7	72.7	94.5	29.13	0.64
Mean ≥0.7	99.6	51.3	95.3	92.5	122.05	0.49	100	76.7	100.0	19.2	∞	0.23	99.6	30.3	86.8	94.0	72.10	0.70
Mean ≥0.8	99.8	46.2	96.8	91.8	184.92	0.54	100.0	74.4	100.0	17.9	∞	0.26	99.8	22.9	89.3	93.4	91.76	0.77

**Score threshold** **(2 contiguous leads with)**	**Alternative outcome definition (AMI with PCI performed) (413 with AMI + PCI, 982 without AMI + PCI)**																	

	**All Patients** **(N = 1,395)**						**STEMI Criteria (+) Patients** **(n = 95)**						**STEMI Criteria (−) Patients** **(n = 1,300)**					
**Spec**	**Sens**	**PPV**	**NPV**	**LR+**	**LR−**	**Spec**	**Sens**	**PPV**	**NPV**	**LR+**	**LR−**	**Spec**	**Sens**	**PPV**	**NPV**	**LR+**	**LR−**
Mean ≥0.5	96.8	26.9	78.2	75.9	8.51	0.76	71.4	80.7	97.3	22.7	2.82	0.27	97.0	12.3	58.0	76.9	4.14	0.90
Mean ≥0.6	97.9	25.2	83.2	75.7	11.77	0.76	71.4	77.3	97.1	20.0	2.70	0.32	98.1	11.1	65.6	76.8	5.68	0.91
Mean ≥0.7	98.9	23.2	89.7	75.4	20.75	0.78	85.7	77.3	98.6	23.0	5.41	0.27	99.0	8.6	73.7	76.5	8.37	0.92
Mean ≥0.8	99.3	21.3	92.6	75.0	30.01	0.79	85.7	75.0	98.5	21.4	5.25	0.29	99.4	6.8	78.6	76.2	10.92	0.94

HATW = hyperacute T-waves; LR+ = positive likelihood ratio; LR− = negative likelihood ratio; NPV = negative predictive value; PCI = percutaneous coronary intervention; PPV = positive predictive value; Sens = sensitivity; Spec = specificity; other abbreviations as in Table 1.

#### HATW clinical outcome analysis

**Figure 5:**
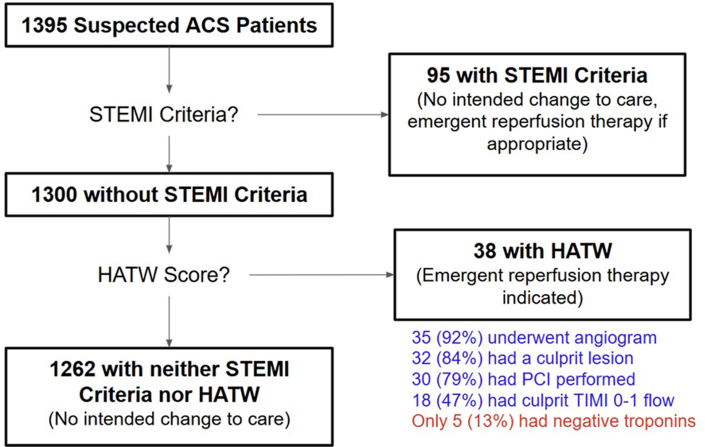
Potential Clinical Effects of HATW Identification in Validation Cohort Flowchart of validation group patients and their clinical results that would have occurred if the HATW score had been applied as a STEMI equivalent finding (after applying STEMI criteria). ACS = acute coronary syndrome; PCI = percutaneous coronary intervention; other abbreviations as in Figures 2 and 3.

For the entire validation cohort (N = 1,395), Figure 5 shows the clinical results that would have occurred if the HATW score had been applied as a STEMI equivalent finding (after applying STEMI criteria). Of the 38 patients with negative STEMI criteria but positive HATW, 32 (84%) had a culprit lesion causing AMI, and only 5 (13%) ruled out for AMI by serial negative troponins.

#### Time to angiography results

OMI patients (TIMI flow grade 0-1 definition) with STEMI criteria were more likely to undergo angiography within 90 minutes of the ECG than OMI patients without STEMI criteria (53/61 = 86.9% vs 14/87 = 16.1%; *P* < 0.001). 10/18 (55.5%) OMI patients without STEMI criteria but with positive HATW score underwent angiography within 90 minutes, with median time to angiography of 1.5 hours (IQR: 0.9-2.5 hours). Time to angiography for each subgroup and each OMI definition is available in Supplemental Table 4.

## Discussion

To our knowledge, this is the first quantifiable definition of HATW to achieve clinically useful diagnostic performance for identifying OMI among possible ACS patients in the ED. Our data support the claim that HATWs cannot be simply defined by T-wave amplitude alone, but instead are better defined by measures of T-wave magnitude (area under the T-wave relative to the QRS amplitude) and T-wave symmetry (position of the T-wave peak relative to the entire T-wave). As such, we recommend that the HATW score components should be measured automatically by software designed for this purpose.

We previously studied 808 high-risk ACS patients in the ED, and we blindly detected HATWs in 49% (72 of 146) of patients who were correctly diagnosed with OMI an average of 3 hours before it was diagnosed by angiography or by STEMI criteria, while maintaining similar specificity.^2^ Similarly, in the DIFOCCULT study, 35 of 282 (12.4%) patients not meeting STEMI criteria were diagnosed with ACO only by the subjective interpretation of HATWs, blinded to outcome.^3^ The results of our HATW score represent initial success at translating subjective expert performance into objective measurements which could be reproduced by others and used in clinical practice by nonexperts.

As a formal STEMI equivalent by the ACC and ESC, we have confirmed high specificity, PPV, and LR+ with reasonable sensitivity to fulfill the role of a STEMI equivalent finding in the population without STEMI criteria. The fact that 58% of patients with HATWs (and without STEMI criteria) underwent angiogram within 90 minutes suggests that some physicians are attempting to follow the guidelines including STEMI equivalent features. On the other hand, 42% of these patients with TIMI flow grade 0-1 culprits did not receive an angiogram within 90 minutes, despite ACC's recommendation for emergent invasive management in such STEMI equivalent patients. This may represent a significant portion of real-world OMI patients who suffer increased morbidity and mortality due to delays in recognition and treatment.^16^

Our study adds further evidence for the low sensitivity of the STEMI criteria for OMI, even defined as TIMI flow grade 0-1 lesion at the time of angiography. In our data, the sensitivity for STEMI criteria was 41%, similar to all other applicable studies.^2^^,^^17^^,^^18^

Many authors contend, without direct evidence, that HATWs are a “precursor” to ST-segment elevation. The implied hope is that the clinician need not spend time and effort to identify HATWs because the OMI patient with HATWs will soon manifest obvious ST-segment elevation on subsequent serial ECGs. In addition to lack of any evidence to support this, and the harm of delay while waiting, we have published a group of 17 patients with TIMI flow grade 0 culprit left anterior descending lesions who had diagnostic HATWs on the first ECG, and none of them developed diagnostic ST-segment elevation on any of the subsequent repeat ECGs.^19^ While we eagerly await further data on this subject, we recommend that ST-segment elevation not be relied upon as a confirmatory finding or delayed surrogate of HATWs in the appropriate clinical context.

Unfortunately, we have shown in our data that potentially simpler measurements of HATWs (such as T-wave amplitude alone) are suboptimal to capture the electrocardiographic appearance and diagnostic value of HATWs. Our data suggest that there is not yet any measure of HATW simple enough for an untrained, nonexpert human to measure by hand with high diagnostic performance. Our HATW score should be measured automatically by computer using the methods described above.

### Study Limitations

This is a retrospective chart review study with the inherent limitations. The study population also represents a high-risk ACS cohort with 10.6% prevalence of OMI, meaning that the results may not be applicable to all-comer ED patients with a lower prevalence of ACS. As with the STEMI criteria and other STEMI equivalent ECG findings, it is of critical importance to apply the HATW score on a clinically appropriate patient population with significant suspicion of ACS after a thorough history and physical exam by an experienced clinician.

Detailed race and ethnicity data were not uniformly available across all sites, limiting our ability to stratify or adjust diagnostic performance across demographic groups. Given that prior work has demonstrated racially distinct ECG morphologies in healthy individuals (eg, greater T-wave amplitude in Black adults), the generalizability of our findings to underrepresented populations warrants further study.

Although T-wave magnitude and symmetry functioned well to identify expert-annotated HATWs in our data, it is likely that these measures do not capture all elements of morphology that are used by ECG experts to optimally identify HATWs. We believe that experts are also able to incorporate other aspects (eg, T-wave concavity features, reciprocal findings in opposite leads including ST-segment depression and T-wave inversion called “reciprocal negative hyperacute T-waves”) which could be the subject of future study. Furthermore, optimally identifying a T-wave as pathologically “symmetric” or “broad” often requires a comparison with prior ECG recordings—a comparison that is frequently unfeasible in acute settings and not incorporated in our present study. Consequently, a T-wave may appear more symmetric at the time of presentation relative to its baseline morphology, even though it does not truly exhibit symmetry (ie, equal intervals from T onset to peak and from peak to end).

Our HATW model using T-wave magnitude and symmetry was not primarily designed to identify the small subset of HATW known as de Winter T-waves (which feature ST depression in the same leads as the increased T-wave magnitude and symmetry). To address this limitation, we reproduced the same methods as above but also included a third score: ST-segment depression. ST-segment depression helps to directly identify leads with de Winter HATWs, and also helps as an indicator of reciprocal ST depression which occurs in leads opposite of HATW. For brevity, we have focused on the simpler 2-variable HATW score (using only T-wave magnitude and symmetry) in the manuscript, but results for the 3-variable HATW score (magnitude, symmetry, and depression) are available in the Supplemental Table 5.

It is important to emphasize that, as the HATW score relies on the measurement of the T-wave area, its calculation requires dedicated software. The busy physician in an emergency setting is unlikely to access a website or manually input data into a calculator in the same manner as with traditional risk scores. Consequently, while the objective definition presented here holds promise for improving the early identification of OMI, its most practical application in the current clinical environment may be as a tool for training artificial intelligence (AI) models and developing automated ECG interpretation systems, rather than as a bedside calculation method.

## Conclusions

HATWs can be quantified and defined by increased T-wave magnitude (area under the curve relative to QRS amplitude) and symmetry. Our resulting HATW score shows significant clinical utility as an ECG finding of OMI in patients with and without concomitant STEMI criteria. This objective and quantifiable definition may improve the identification, communication, and inter-rater reliability of HATWs, as well as the explainability of AI models designed to identify ECG features of OMI. While further external validation studies are needed, our findings add further support to the ACC and ESC recommendations that HATW be identified and treated as ECG features of ACO.

Perspectives **COMPETENCY IN MEDICAL KNOWLEDGE:** HATWs are a specific ECG manifestation of ACO MI that can be objectively defined and recognized to fulfill their role as a recommended STEMI equivalent ECG finding, prompting consideration of emergent reperfusion therapy even in the absence of traditional STEMI millimeter criteria.

**TRANSLATIONAL OUTLOOK:** This is the first study using an objective definition of HATW with clinically meaningful performance for emergently identifying ACO. External validation and clinical implementation studies are needed. The measurements and HATW formula should be integrated into ECG machines and/or ECG viewing software for feasibility of use.

## Funding support and author disclosures

The work performed by authors and scientists for this publication was in part funded by Powerful Medical. Drs Meyers, Simančík, Herman, Rafajdus, and Smith have financial relationships with Powerful Medical. Dr Herman is the Chief Medical Officer of Powerful Medical. Drs Simančík and Herman are employees of Powerful Medical. Dr Meyers is a consultant to Powerful Medical. Drs Meyers and Smith hold stock options in Powerful Medical. All other authors have reported that they have no relationships relevant to the contents of this paper to disclose.
