# Supplementary material for: Hyperacute T Waves Are Specific for Occlusion Myocardial Infarction, Even Without Diagnostic ST-Segment Elevation
Source: JACC Adv. 2025 Aug 31;4(10):102120. doi: 10.1016/j.jacadv.2025.102120 (PMC12791876; doi:10.1016/j.jacadv.2025.102120)

Supplemental Figure 1: Example ECG in derivation data marked as containing hyperacute T waves in leads II, III, and aVF.


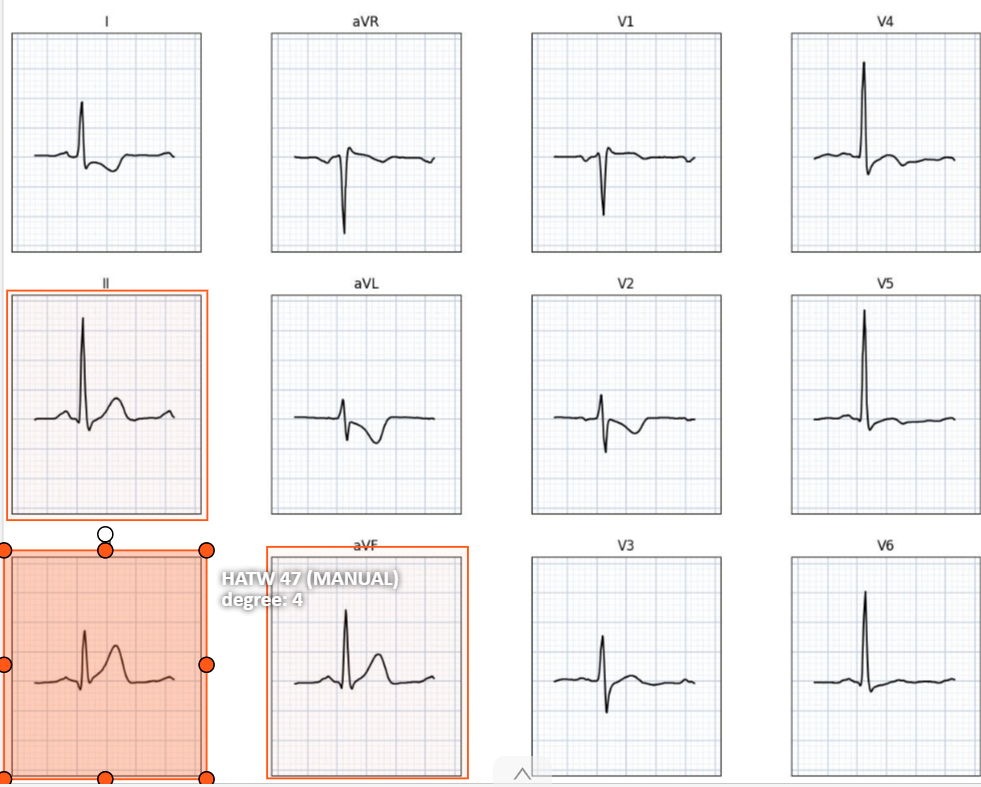


Supplemental Figure 2: HATW Model architecture.


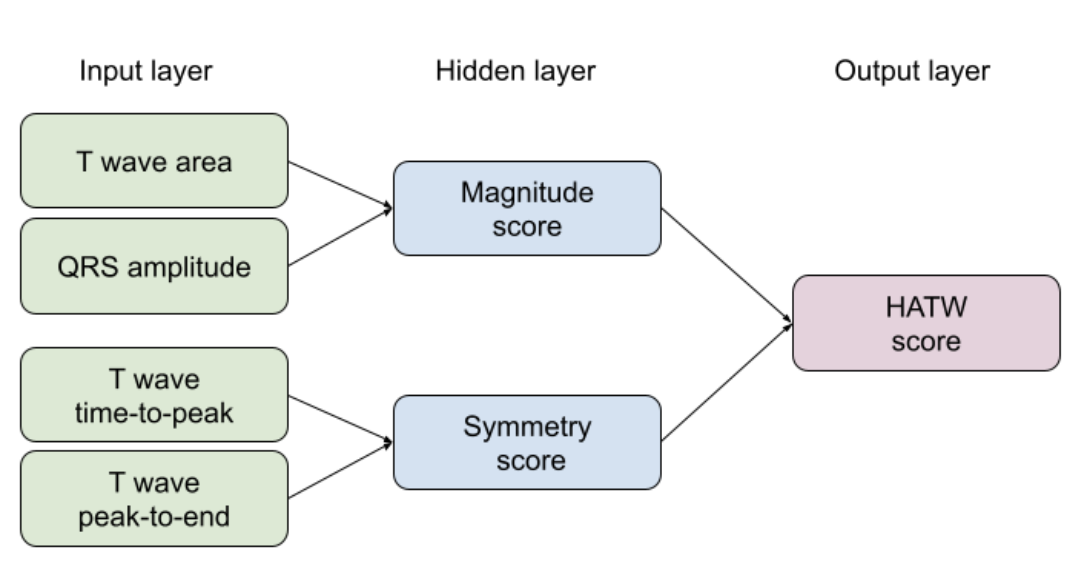


Supplemental Figure 3: Interrater reliability measurements between HPM and SWS for HATWs in individual leads. Overall, the two experts had 90% (3504/3894) agreement on the presence/absence of HATWs. Of the 390 disagreements, 220 (56%) occurred when one expert chose degree 0 (not hyperacute) and the other chose degree 1 (barely considered hyperacute, usually only identifiable based on surrounding/reciprocal context available in other leads).


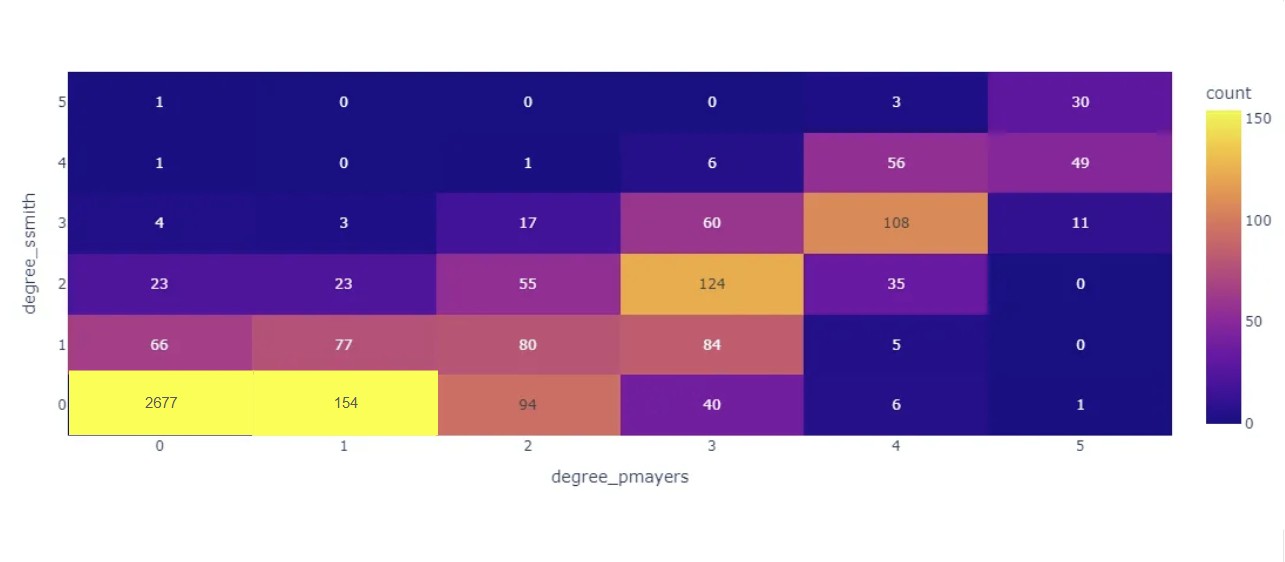


Supplemental Figure 4. Individual leads from ECGs A and B from Figures 3 and 4 are presented on magnitude, symmetry, and HATW score plots. The red line represents the threshold score of 0.5 for each logistic regression. Two contiguous leads with mean HATW score 0.7 are considered positive for HATW in our study.


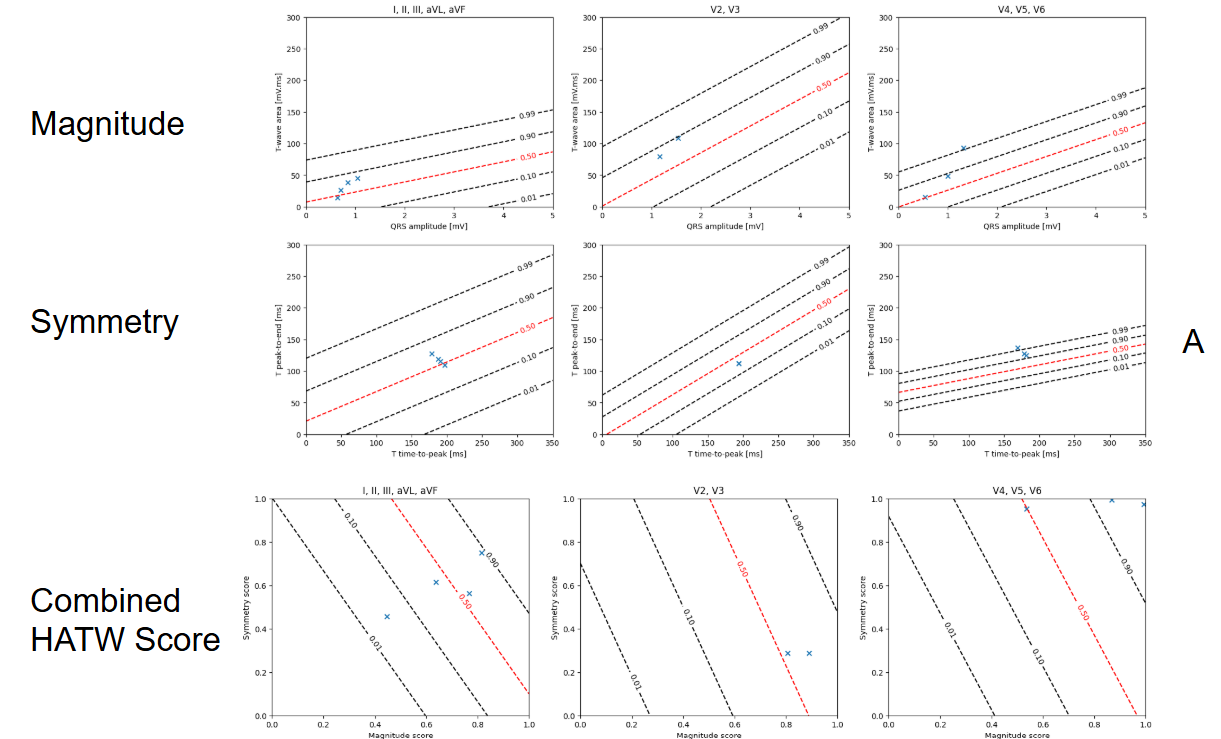


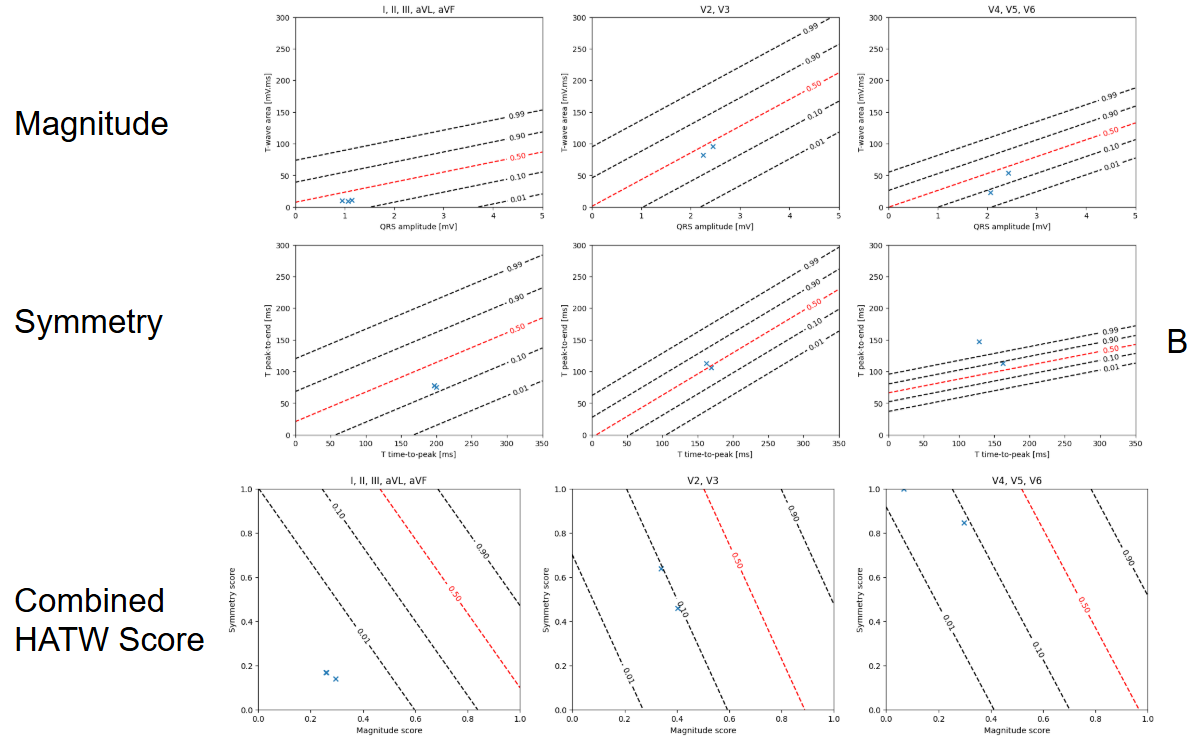


Supplemental Table 1: Number of hyperacute and control (normal or non-hyperacute) T waves in each lead in the derivation group.

| I  79 HATW  1642 Control | aVR  0 HATW  1642 Control | V1  38 HATW  1642 Control | V4  162 HATW  1642 Control |
| --- | --- | --- | --- |
| II  165 HATW  1642 Control | aVL  60 HATW  1642 Control | V2  145 HATW  1642 Control | V5  143 HATW  1642 Control |
| III  166 HATW  1642 Control | aVF  169 HATW  1642 Control | V3  178 HATW  1642 Control | V6  96 HATW  1642 Control |

Supplemental Table 2: Comparison of relevant T wave amplitude, T wave area, and T wave magnitude for HATWs versus control group T waves, for each lead group (limb leads, V2-3, V4-6).

| Lead Group |  | Control Leads  (average value) | HATW Leads  (average value) | Pearson Correlation | Optimal F1 Score | ROC AUC | Sensitivity at 98% Specificity | Threshold for 98% Specificity |
| --- | --- | --- | --- | --- | --- | --- | --- | --- |
| Limb Leads | T wave amplitude | 0.22 mV | 0.35 mV | 0.31 | 0.31 | 0.76 | 9% | 0.50 mV |
|  | T wave area | 16.63 mV*ms | 42.23 mV*ms | 0.48 | 0.44 | 0.86 | 23% | 48 mV*ms |
|  | T wave area / QRS amplitude | 22.60 ms | 69.03 ms | 0.52 | 0.42 | 0.86 | 24% | 71 ms |
|  |  |  |  |  |  |  |  |  |
|  |  | Control Leads  (average value) | HATW Leads  (average value) | Pearson Correlation | Optimal F1 Score | ROC AUC | Sensitivity at 98% Specificity | Threshold for 98% Specificity |
| V2-V3 | Isolated T wave amplitude | 0.35 mV | 0.59 mV | 0.27 | 0.25 | 0.72 | 9% | 0.89 mV |
|  | T wave area | 34.40 mV*ms | 69.43 mV*ms | 0.32 | 0.26 | 0.75 | 11% | 103 mV*ms |
|  | T wave area / QRS amplitude | 27.63 ms | 79.43 ms | 0.51 | 0.41 | 0.84 | 23% | 81 ms |
|  |  |  |  |  |  |  |  |  |
|  |  | Control Leads  (average value) | HATW Leads  (average value) | Pearson Correlation | Optimal F1 Score | ROC AUC | Sensitivity at 98% Specificity | Threshold for 98% Specificity |
| V4-V6 | Isolated T wave amplitude | 0.26 mV | 0.47 mV | 0.31 | 0.26 | 0.78 | 13% | 0.62 mV |
|  | T wave area | 20.24 mV*ms | 50.02 mV*ms | 0.39 | 0.31 | 0.83 | 19% | 58 mV*ms |
|  | T wave are / QRS amplitude | 17.17 ms | 46.73 ms | 0.48 | 0.46 | 0.89 | 32% | 44 ms |

Supplemental ata Table 3: Diagnostic accuracy statistics of all combinations of HATW score cutoff, and presence of STEMI criteria, for each outcome definition studied.

| **Primary OMI Definition (AMI with Culprit lesion with TIMI 0-1 Flow)** | | | | | | | | | | | | | | | | | | | | | |
| --- | --- | --- | --- | --- | --- | --- | --- | --- | --- | --- | --- | --- | --- | --- | --- | --- | --- | --- | --- | --- | --- |
|  | **All Patients**  N=1,395 | | | | | | | **STEMI Criteria (+) Patients**  N=95 | | | | | | | **STEMI Criteria (-) Patients**  N=1300 | | | | | | |
| Score Threshold  (2 contiguous leads with:) | Spec | Sens | PPV | NPV | LR+ | LR- | Spec | | Sens | PPV | NPV | LR+ | LR- | Spec | | Sens | PPV | NPV | LR+ | LR- |  |
| Both ≥0.5 | 95.2 | 46.0 | 53.1 | 93.7 | 9.55 | 0.57 | 35.3 | | 80.3 | 69.0 | 50.0 | 1.24 | 0.56 | 96.9 | | 21.8 | 33.3 | 94.5 | 6.98 | 0.81 |  |
| Both ≥0.6 | 96.4 | 43.9 | 59.1 | 93.5 | 12.17 | 0.58 | 41.2 | | 80.3 | 71.0 | 53.9 | 1.37 | 0.48 | 97.9 | | 18.4 | 39.0 | 94.4 | 8.93 | 0.83 |  |
| Both ≥0.7 | 97.2 | 41.9 | 63.9 | 93.4 | 14.91 | 0.60 | 44.1 | | 78.7 | 71.6 | 53.6 | 1.41 | 0.48 | 98.7 | | 16.1 | 46.7 | 94.3 | 12.19 | 0.85 |  |
| Both ≥0.8 | 97.7 | 39.2 | 66.7 | 93.1 | 16.82 | 0.62 | 47.1 | | 73.8 | 71.4 | 50.0 | 1.39 | 0.56 | 99.1 | | 14.9 | 54.2 | 94.2 | 16.42 | 0.86 |  |
| Mean ≥0.5 | 94.2 | 46.6 | 48.6 | 93.7 | 7.97 | 0.57 | 32.4 | | 82.0 | 68.5 | 50.0 | 1.21 | 0.56 | 95.9 | | 21.8 | 27.5 | 94.5 | 5.30 | 0.82 |  |
| Mean ≥0.6 | 95.4 | 46.0 | 54.4 | 93.7 | 10.05 | 0.57 | 38.2 | | 80.3 | 70.0 | 52.0 | 1.30 | 0.51 | 97.0 | | 21.8 | 34.6 | 94.5 | 7.35 | 0.81 |  |
| Mean ≥0.7 | 96.8 | 45.3 | 62.6 | 93.7 | 14.10 | 0.57 | 41.2 | | 80.3 | 71.0 | 53.9 | 1.37 | 0.48 | 98.4 | | 20.7 | 47.4 | 94.5 | 12.54 | 0.81 |  |
| Mean ≥0.8 | 97.4 | 41.9 | 65.3 | 93.4 | 15.81 | 0.60 | 44.1 | | 78.7 | 71.6 | 53.6 | 1.41 | 0.48 | 98.9 | | 16.1 | 50.0 | 94.3 | 13.99 | 0.85 |  |
| **Alternative OMI Definition (AMI with Culprit lesion with TIMI 0-1 Flow, or TIMI 2-3 Flow with Troponin T > 1,000 ng/L)** | | | | | | | | | | | | | | | | | | | | | |
|  | **All Patients**  N=1,395 | | | | | | | **STEMI Criteria (+) Patients**  N=95 | | | | | | | **STEMI Criteria (-) Patients**  N=1300 | | | | | | |
| Score Threshold  (2 contiguous leads with:) | Spec | Sens | PPV | NPV | LR+ | LR- | Spec | | Sens | PPV | NPV | LR+ | LR- | Spec | | Sens | PPV | NPV | LR+ | LR- |  |
| Both ≥0.5 | 96.8 | 40.6 | 71.1 | 89.5 | 12.85 | 0.61 | 50.0 | | 80.5 | 87.3 | 37.5 | 1.61 | 0.39 | 97.6 | | 19.7 | 50.9 | 90.5 | 8.12 | 0.82 |  |
| Both ≥0.6 | 97.7 | 37.1 | 75.5 | 89.0 | 16.04 | 0.64 | 55.6 | | 79.2 | 88.4 | 38.5 | 1.78 | 0.37 | 98.4 | | 15.0 | 53.7 | 90.1 | 9.07 | 0.86 |  |
| Both ≥0.7 | 98.5 | 35.3 | 81.4 | 88.8 | 22.90 | 0.66 | 61.1 | | 77.9 | 89.6 | 39.3 | 2.00 | 0.36 | 99.1 | | 12.9 | 63.3 | 89.9 | 13.61 | 0.88 |  |
| Both ≥0.8 | 98.8 | 32.6 | 83.9 | 88.5 | 27.16 | 0.68 | 61.1 | | 72.7 | 88.9 | 34.4 | 1.87 | 0.45 | 99.4 | | 11.6 | 70.8 | 89.8 | 18.95 | 0.89 |  |
| Mean ≥0.5 | 95.8 | 41.5 | 65.5 | 89.6 | 9.93 | 0.61 | 50.0 | | 83.1 | 87.7 | 40.9 | 1.66 | 0.34 | 96.5 | | 19.7 | 42.0 | 90.4 | 5.69 | 0.83 |  |
| Mean ≥0.6 | 96.9 | 39.7 | 71.2 | 89.4 | 12.94 | 0.62 | 50.0 | | 79.2 | 87.1 | 36.0 | 1.58 | 0.42 | 97.7 | | 19.1 | 50.9 | 90.4 | 8.14 | 0.83 |  |
| Mean ≥0.7 | 98.1 | 38.0 | 79.4 | 89.2 | 20.19 | 0.63 | 55.6 | | 79.2 | 88.4 | 38.5 | 1.78 | 0.37 | 98.8 | | 16.3 | 63.2 | 90.3 | 13.50 | 0.85 |  |
| Mean ≥0.8 | 98.6 | 35.3 | 83.2 | 88.9 | 25.74 | 0.66 | 61.1 | | 77.9 | 89.6 | 39.3 | 2.00 | 0.36 | 99.2 | | 12.9 | 67.9 | 89.9 | 16.58 | 0.88 |  |
| **Alternative OMI Definition (Expert Chart Review)** | | | | | | | | | | | | | | | | | | | | | |
|  | **All Patients**  N=1,395 | | | | | | | **STEMI Criteria (+) Patients**  N=95 | | | | | | | **STEMI Criteria (-) Patients**  N=1300 | | | | | | |
| Score Threshold  (2 contiguous leads with:) | Spec | Sens | PPV | NPV | LR+ | LR- | Spec | | Sens | PPV | NPV | LR+ | LR- | Spec | | Sens | PPV | NPV | LR+ | LR- |  |
| Both ≥0.5 | 98.4 | 54.8 | 85.2 | 92.9 | 34.45 | 0.46 | 80.0 | | 77.8 | 98.6 | 16.7 | 3.89 | 0.28 | 98.5 | | 35.8 | 68.4 | 94.4 | 23.70 | 0.65 |  |
| Both ≥0.6 | 99.3 | 50.8 | 91.8 | 92.4 | 67.67 | 0.50 | 100 | | 76.7 | 100 | 19.2 | ∞ | 0.23 | 99.2 | | 29.4 | 78.1 | 93.9 | 38.63 | 0.71 |  |
| Both ≥0.7 | 99.7 | 46.7 | 95.9 | 91.8 | 141.61 | 0.53 | 100 | | 74.4 | 100.0 | 17.9 | ∞ | 0.26 | 99.7 | | 23.9 | 86.7 | 93.5 | 70.15 | 0.76 |  |
| Both ≥0.8 | 99.9 | 43.2 | 98.9 | 91.4 | 540.25 | 0.57 | 100.0 | | 70.0 | 100.0 | 15.6 | ∞ | 0.30 | 99.9 | | 21.1 | 95.8 | 93.3 | 263.75 | 0.79 |  |
| Mean ≥0.5 | 97.7 | 57.3 | 80.3 | 93.2 | 24.48 | 0.44 | 80.0 | | 80.0 | 98.6 | 18.2 | 4.00 | 0.25 | 97.7 | | 38.5 | 60.9 | 94.6 | 16.97 | 0.63 |  |
| Mean ≥0.6 | 98.7 | 54.8 | 87.2 | 92.9 | 40.87 | 0.46 | 80.0 | | 76.7 | 98.6 | 16.0 | 3.83 | 0.29 | 98.7 | | 36.7 | 72.7 | 94.5 | 29.13 | 0.64 |  |
| Mean ≥0.7 | 99.6 | 51.3 | 95.3 | 92.5 | 122.05 | 0.49 | 100 | | 76.7 | 100.0 | 19.2 | ∞ | 0.23 | 99.6 | | 30.3 | 86.8 | 94.0 | 72.10 | 0.70 |  |
| Mean ≥0.8 | 99.8 | 46.2 | 96.8 | 91.8 | 184.92 | 0.54 | 100.0 | | 74.4 | 100.0 | 17.9 | ∞ | 0.26 | 99.8 | | 22.9 | 89.3 | 93.4 | 91.76 | 0.77 |  |
| **Alternative OMI Definition (AMI with PCI Performed)** | | | | | | | | | | | | | | | | | | | | | |
|  | **All Patients**  N=1,395 | | | | | | | **STEMI Criteria (+) Patients**  N=95 | | | | | | | **STEMI Criteria (-) Patients**  N=1300 | | | | | | |
| Score Threshold  (2 contiguous leads with:) | Spec | Sens | PPV | NPV | LR+ | LR- | Spec | | Sens | PPV | NPV | LR+ | LR- | Spec | | Sens | PPV | NPV | LR+ | LR- |  |
| Both ≥0.5 | 97.6 | 22.0 | 82.8 | 70.2 | 9.11 | 0.80 | 66.7 | | 77.5 | 97.2 | 16.7 | 2.33 | 0.34 | 97.8 | | 9.4 | 68.4 | 94.4 | 4.25 | 0.93 |  |
| Both ≥0.6 | 98.6 | 20.1 | 88.2 | 70.0 | 14.04 | 0.81 | 83.3 | | 76.4 | 98.6 | 19.2 | 4.58 | 0.28 | 98.7 | | 7.4 | 78.1 | 93.9 | 5.58 | 0.94 |  |
| Both ≥0.7 | 99.1 | 18.4 | 91.8 | 69.7 | 20.94 | 0.82 | 83.3 | | 74.2 | 98.5 | 17.9 | 4.45 | 0.31 | 99.2 | | 5.8 | 86.7 | 93.5 | 7.58 | 0.95 |  |
| Both ≥0.8 | 99.5 | 17.0 | 94.3 | 69.3 | 30.87 | 0.83 | 83.3 | | 69.7 | 98.4 | 15.6 | 4.18 | 0.36 | 99.6 | | 5.1 | 95.8 | 93.3 | 11.55 | 0.95 |  |
| Mean ≥0.5 | 96.9 | 23.6 | 80.3 | 70.6 | 7.69 | 0.79 | 66.7 | | 79.8 | 97.3 | 18.2 | 2.39 | 0.30 | 97.1 | | 10.9 | 60.9 | 94.6 | 3.80 | 0.92 |  |
| Mean ≥0.6 | 97.8 | 21.7 | 84.0 | 70.2 | 9.93 | 0.80 | 66.7 | | 76.4 | 97.1 | 16.0 | 2.29 | 0.35 | 98.0 | | 9.4 | 72.7 | 94.5 | 4.72 | 0.92 |  |
| Mean ≥0.7 | 98.9 | 20.1 | 90.7 | 70.0 | 18.25 | 0.81 | 83.3 | | 76.4 | 98.6 | 19.2 | 4.58 | 0.28 | 99.0 | | 7.4 | 86.8 | 94.0 | 7.43 | 0.94 |  |
| Mean ≥0.8 | 99.2 | 18.2 | 92.6 | 69.6 | 23.66 | 0.82 | 83.3 | | 74.2 | 98.5 | 17.9 | 4.45 | 0.31 | 99.3 | | 5.6 | 89.3 | 93.4 | 8.45 | 0.95 |  |

Supplemental Table 4: Time to angiogram with respect to STEMI criteria and HATW subgroups.


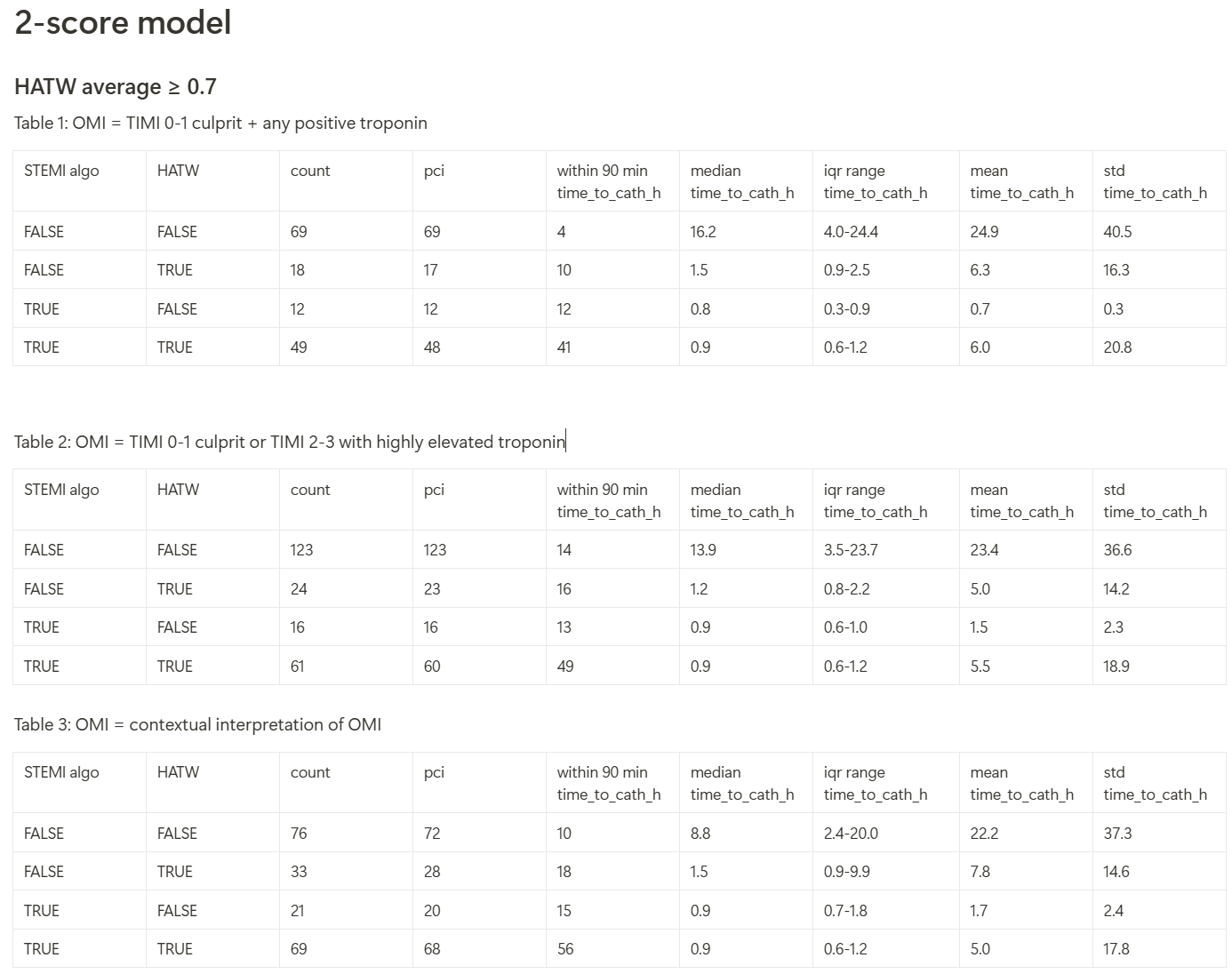


Supplemental Table 5: Results using the 3-Variable HATW Score (Magnitude, Symmetry, and ST Depression)


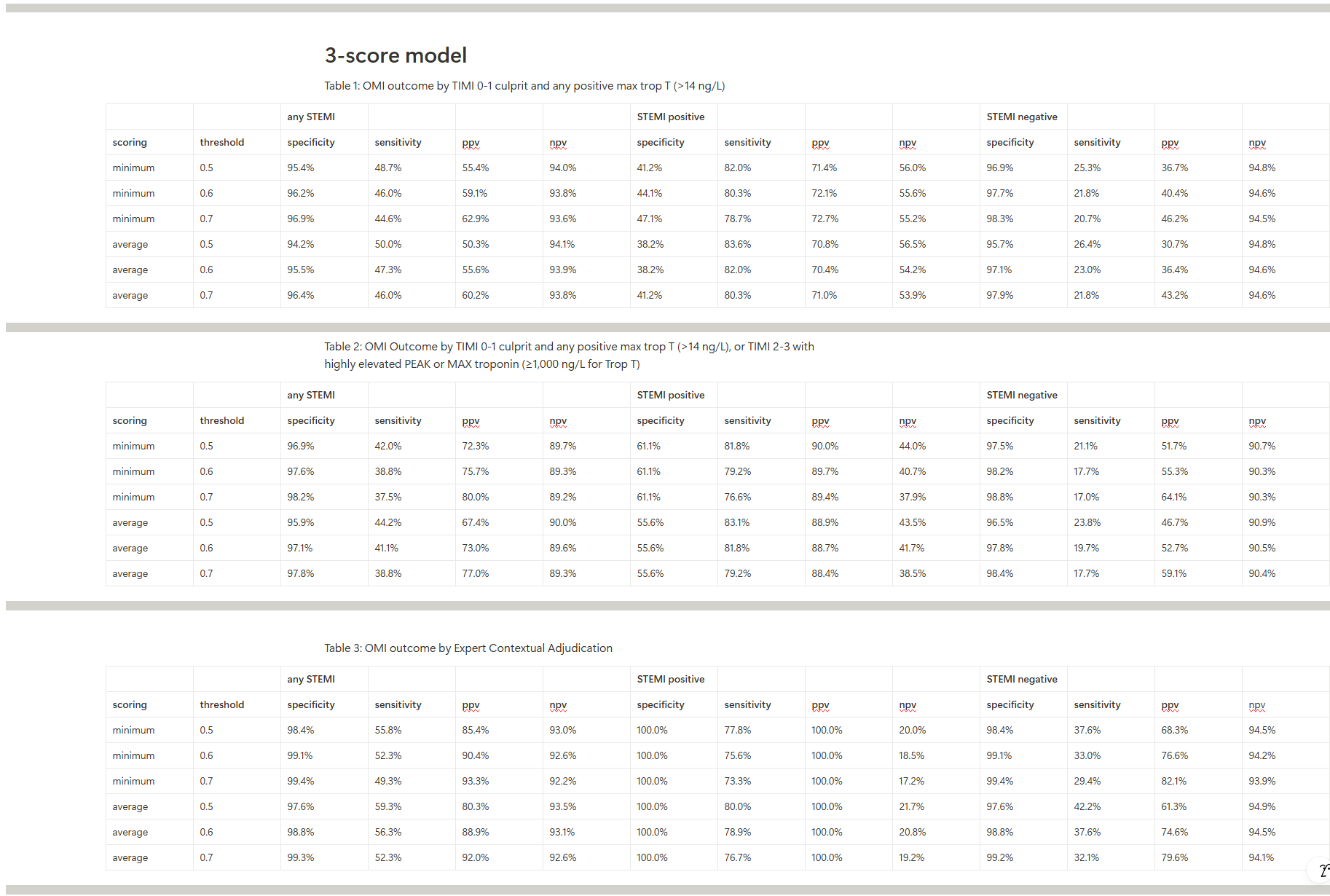

Supplement: Supplemental Figures 1, 2, 3, and 4, and Supplemental Tables 1, 2, 3, 4, and 5 [file mmc1.docx]
